# Diagnosis of Basal Cell Carcinoma by Reflectance Confocal Microscopy: Study Design and Protocol of a Randomized Controlled Multicenter Trial

**DOI:** 10.2196/resprot.5757

**Published:** 2016-06-30

**Authors:** Malou Peppelman, Kim P Nguyen, Hans A.C Alkemade, Birgitte Maessen-Visch, Jan C.M Hendriks, Piet E.J van Erp, Eddy M.M Adang, Marie-Jeanne P Gerritsen

**Affiliations:** ^1^ Radboud University Medical Center Department of Dermatology Nijmegen Netherlands; ^2^ Canisius Wilhelmina Hospital Department of Dermatology Nijmegen Netherlands; ^3^ Rijnstate hospital Department of Dermatology Arnhem-Velp Netherlands; ^4^ Radboud University Medical Center Department for Health Evidence Nijmegen Netherlands

**Keywords:** basal cell carcinoma, reflectance confocal microscopy, diagnosis, cost effectiveness

## Abstract

**Background:**

Skin cancer, including basal cell carcinoma (BCC), has become a major health care problem. The limitations of a punch biopsy (at present the gold standard) as diagnostic method together with the increasing incidence of skin cancer point out the need for more accurate, cost-effective, and patient friendly diagnostic tools. In vivo reflectance confocal microscopy (RCM) is a noninvasive imaging technique that has great potential for skin cancer diagnosis.

**Objective:**

To investigate whether in vivo RCM can correctly identify the subtype of BCC and to determine the cost-effectiveness of RCM compared with punch biopsy (usual care). Study design: Randomized controlled multicenter trial.

**Methods:**

On the basis of 80% power and an alpha of 0.05, 329 patients with lesions clinically suspicious for BCC will be included in this study. Patients will be randomized for RCM or for a punch biopsy (usual care). When a BCC is diagnosed, surgical excision will follow and a follow-up visit will be planned 3 months later. Several questionnaires will be filled in (EQ-5D, EQ-5D VAS, iMTA PCQ, and TSQM-9). We will perform statistical analysis, cost-effectiveness, and patient outcome analysis after data collection.

**Results:**

This research started in January 2016 and is ethically approved. We expect to finish this study at the end of 2018.

**Conclusions:**

In this study, we will investigate whether RCM is at least as good in identifying BCC subtypes as conventional pathological investigation of skin biopsies. Anticipating that RCM is found to be a cost-effective alternative, it saves on direct medical consumption like labor of the pathologist and other medical personnel as well as materials related to treatment failure with at least equal effectiveness.

**Trial Registration:**

Clinicaltrials.gov NCT02623101; https://clinicaltrials.gov/ct2/show/NCT02623101 (Archived by WebCite at http://www.webcitation.org/6id54WQa2)

## Introduction

### Skin Cancer

Skin cancer is a common type of cancer and its incidence is increasing rapidly in Western countries [1-3]. This cancer comprises two types: melanoma (MM) and nonmelanoma skin cancer (NMSC). NMSC is further divided into basal cell carcinoma (BCC), squamous cell carcinoma (SCC), and its precursors actinic keratosis (AK) and Bowen disease. In the Netherlands, the registry of NMSC is poor. However, based on recent literature and guidelines, it is estimated that the incidence of malignant skin tumors and the premalignant AK is around 235,000 in 2015. This will have a major impact on our health care system. Moreover, it is predicted that numbers will rise at the rate of 4.5-8% per year, depending on the type of skin cancer. Currently, in case of suspicion of NMSC, the pathological examination of a punch biopsy is the gold standard, according to the Dutch guidelines. In case of clinical suspicion of AK, the diagnosis is made *à vue*, without pathological confirmation. Already in 2003 in the United States, skin cancer was found to be among the most costly of all cancers to treat. Therefore, it is evident that skin cancer places an enormous burden on health care systems with increasing costs [4]. In case of suspicion of skin cancer, it is important to diagnose and treat it in an early phase, preferably in a patient friendly manner. As BCC is the most common skin cancer (about 75% of all skin cancers), this study will focus on studying this skin cancer type. Clinically, BCC can vary in appearance but is often characterized by small, translucent, or pearly papules, with telangiectasias [5]. In the past, the diagnosis was mainly made clinically. However, noninvasive therapies have become available; therefore, determination of the BCC subtype has become more important. For this reason, pathological analysis of a punch biopsy is currently the gold standard to confirm the clinical diagnosis and determine the subtype of BCC. The following subtypes of BCC can be distinguished: superficial (sBCC), nodular (nBCC), aggressive BCC (micronodular (mnBCC), and infiltrative (iBCC)) [6]. It is experienced that there is a sample error in 29% of the cases with the conventional diagnostic procedure, resulting in an incorrect subtype diagnosis [7]. For this reason and because of the increasing incidence of skin cancer, more accurate, cost-efficient, and patient friendly diagnostic tools are desirable.

### Reflectance Confocal Microscopy

Reflectance confocal microscopy (RCM) is a noninvasive imaging technique. It provides real-time images of cell and tissue structures and in vivo dynamics, without the need for ex vivo tissue samples. RCM visualizes human skin up to a depth of around 250 μm [8-12]. Refractive index differences between cells and surrounding tissue provide the contrast. The contrast of RCM imaging of the skin is mainly provided by melanin and keratin [10]. Most, but not all, tumors can be visualized. For thicker tumors, RCM may help to find the optimal localization to perform a punch biopsy, as superficial features in these tumors may help to spot these lesions [13]. Moreover, RCM can image the whole tumor. RCM features for NMSC have been described that showed a high correlation with conventional pathological features [13-15]. These features allow diagnosing AK, SCC, and BCC [13-15]. For both the BCC subtypes, nodular and micronodular BCC, the following RCM characteristics are described: tumor nests with peripheral palisading, branch-like structures, fibrotic septa, and increase of vascular diameter. The size and shape of the tumor nests allows further distinction between these BCCs. Solar elastosis and tumor nests connected with the basal cell layer characterize superficial BCC [14]. iBCCs are more challenging to visualize due to their histological complex appearance and deeper location [16].

Only few studies report data about diagnostic accuracy of RCM for primary BCC diagnosis [14,16-19]. These studies show a high sensitivity and specificity for RCM as diagnostic tool for BCC. Although these show the potential of RCM in BCC diagnosis, prospective large-scale studies are lacking. In addition to BCC diagnosis by RCM in general, no diagnostic accuracy data were reported on determination of BCC subtype by RCM. Such studies are required for implementation of RCM in the routine patient care and incorporation into the health insurance system. Implementation of RCM in the routine patient care settings has the advantage of making a diagnosis at the first consultation and therefore, the patient can be treated at short term. A second consultation for explaining the diagnosis and performing the treatment might be unnecessary. Therefore, time saved by using the RCM can be used for other new patients.

### Objectives

The primary objective of this study is to investigate whether in vivo RCM can identify the subtype of BCC at least as correctly as a skin punch biopsy. We are hypothesizing that RCM imaging allows correct identification of the BCC subtype (nBCC, mnBCC, sBCC, iBCC, and mixed type BCC), and true and false positive results are equal or better as compared with conventional pathological investigation of skin biopsies (gold standard). It is postulated that RCM is more cost-effective and patient friendly compared with the current procedure. Therefore, the quality of life (Qol), costs, and quality adjusted life years (QALYs) will be evaluated as the secondary outcome measures. Overall, with the implementation of RCM in dermatology skin cancer care, it is aimed to contribute to cost-effective, noninvasive, patient friendly diagnostics.

## Methods

### Recruitment, Inclusion, and Study Design

Patients with lesions clinically suspicious (diagnosis *à vue*) for BCC, eligible for RCM, visiting the dermatological departments of the Radboud University Medical Center, Nijmegen, the Canisius Wilhelmina Hospital, Nijmegen, and the Rijnstate Hospital Arnhem-Velp, in The Netherlands will be asked to join this study.

In order to be eligible for participation in this study, a subject must meet all of the following criteria:

- Patients must be 18 years and above.
- Patients must be able to adhere to all requirements of the study.
- Patients must be willing to give written informed consent.
- There must be clinical diagnosis/clinical suspicion of basal cell carcinoma.

A potential subject who meets any of the following criteria will be excluded from participation in this study:

- A patient participating in other investigational research currently or in the previous 28 days before the study
- Patient having a medical condition which excludes participating the research, according to the investigator
- Incapacitated subjects
- Subjects with lesion(s) on parts of the body which do not allow adequate imaging of the tumor with RCM

**Figure 1:**
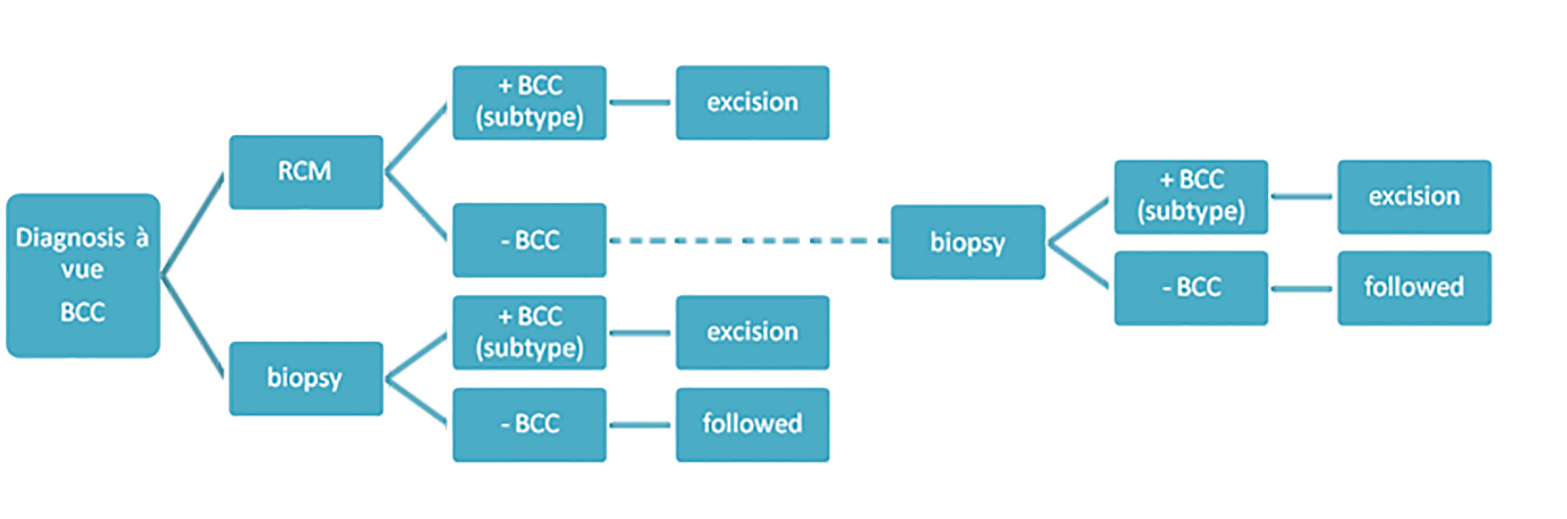
Scheme of randomization. Two randomization arms are designed. After inclusion, a patient with a clinical diagnosis of BCC (diagnosis à vue) will be randomized over the two arms. One arm contains the standard procedure of a biopsypunch biopsy, the other arm contains the diagnostic tool to be investigated: RCM. A punch biopsy will also be obtained when there is no suspicion of a BCC using RCM.

When a patient meets these criteria and gives informed consent, he or she is assigned to a randomization arm according to a computer-generated block randomization (Castor) (Figure 1).

### Power Calculation

The primary outcome in this study is the percentage of correctly identified subtype of confirmed BCC after excision (gold standard in this study). We assume that this is 71% when a punch biopsy is used and 85% when RCM is used (based on an ongoing study). In this case, 148 patients are needed per group to obtain a power of 80% (Fisher’s-exact, two-sided, alpha=0.05). We expect that 10% of the patients with a clinically suspected BCC will not have histopathologically confirmed BCC. Therefore, we will include approximately 329 patients with a clinical suspicion of BCC. In this multicenter randomized controlled trial (RCT), it is also possible to obtain empirical estimates of the (cost-) effectiveness in daily clinical practice, beyond the diagnostic value. The expected benefit of the experimental diagnostic tool is anticipated at €92 per patient. On the basis of a conservative choice of the SD of €100 and the CI of 95%, 146 patients per group are required. Counting a 10% possible dropouts, around 322 patients need to be included.

To answer both questions, in total 329 patients suspected with a clinical suspected BCC will be included in this study.

### Outcome Measures

The primary outcome measure is defined as correct subtyping of the BCC after excision. The histopathological diagnosis of the excision specimen will be compared with either the diagnosis made by RCM or a punch biopsy. Secondary outcome measures are Qol, Cost, and QALYs.

### Procedure

Patients will be assigned to either the RCM or the punch biopsy study arm (Figure 1). When a BCC is diagnosed using RCM or the gold standard (punch biopsy), surgical excision will follow according to standard care-time schedule at the center where the BCC is diagnosed with margins according to the guidelines (3 mm for sBCC and nBCC, 5 mm for aggressive BCC). In case of a BCC, a follow-up visit will be planned 3 months after surgery. If the diagnosis reveals the absence of a BCC, the patients will again be followed-up after 3 months. During visit 1 (diagnostic procedure) several questionnaires will be filled in (EQ-5D, EQ-5d VAS, iMTA PCQ, and TSQM-9). At the follow-up visit after treatment, the questions about satisfaction of the diagnostic procedure will be asked again. In order to establish the added monetary value of RCM, a contingent valuation method (CVM) was used. Patients that belong to the RCM arm and also had a punch biopsy, in which both diagnosis had the absence of a BCC, were interviewed according to the CVM.

RCM will be performed with the commercially available Vivascope 1500 (Caliber imaging & diagnostics, Rochester, NY, USA) according to a standardized protocol. Vivablocks of 4 × 4 mm will be made at the level of the stratum corneum, stratum spinosum, dermal epidermal junction, and dermis in order to find RCM features for BCC and the subtype. Vivastacks will be made in the areas of interest. Movies will be made to document vascularization. When indicated, the Vivascope 3000 handheld device will be used. The RCM user is working for 4 years with the device. If a punch biopsy needs to be obtained according to the randomization scheme, this will occur after local anesthesia (1% xylocaine/adrenaline) and the punch biopsy will have a diameter of 3 mm. The punch biopsy will be taken from the most clinically suspected area of the lesion.

### Analysis

After data collection, analyses will be performed. The Fisher’s-exacts test will be used to test the differences in the primary outcome between the two study arms (biopsy, RCM) for statistical significance. Multivariable logistic regression will be used to study possible differences between the subtypes and the effect of possible other variables. This will be done in order to evaluate variables or sets of variables for its discriminative character that can be used to make protocols and guidelines for future Dutch RCM users.

### Cost-Effectiveness Analysis

The cost analysis comprises two main parts. First, on patient level, volumes of care will be measured prospectively over the time path of the clinical trial using the iMCQ (a generic instrument for measuring medical costs [20]) complemented with procedure specific cost information like cost of RCM equipment and patient out-of-pocket expenses, such as over-the-counter drugs (for example pain related). Relevant, (missing) entries will be verified or completed by data from the medical records or inpatient treatment facility’s administration system. Second, per modality (RCM or usual care) standard cost prices will be determined using the Dutch guideline [21] or else real/full cost prices via activity-based costing. Productivity losses will be estimated using a patient-based questionnaire [22]. The friction-cost method will be applied following the Dutch guidelines [21].

### Patient Outcome Analysis

The effect analysis adheres to the design of a superiority/equivalent RCT and measures diagnostic performance and Qol at baseline, and at fixed points along the follow-up of the RCT. To measure the quality of the health status of the patients, a validated so-called health-related quality of life (HRQoL) instrument will be used, the EuroQol-5D-3L (EQ-5D) [23]. This HRQoL instrument will be completed by the patients and is available in a validated Dutch translation [24]. The EQ-5D is a generic HRQoL instrument comprising five domains: mobility, self-care, usual activities, pain/discomfort, and anxiety/depression. The EQ-5D index is obtained by applying predetermined weights to the five domains. This index gives a societal-based global quantification of the patient’s health status on a scale ranging from 0 (death) to 1 (perfect health). Patients will also be asked to rate their overall HRQoL on a visual analogue scale (EQ-5D VAS) consisting of a line ranging from 0 (worst imaginable health status) to 100 (best imaginable). The patient outcome analysis will be complemented with a CVM questionnaire and measures of satisfaction and pain related to diagnosing subtype BCC.

## Results

This investigator initiated multicenter RCT is conducted according to the principles of the Declaration of Helsinki (2013) and in accordance with the medical Research Involving Human Subjects Act (WMO). The study is funded by ZonMw, a Dutch organization that finances health science, and thereby stimulates the use of obtained knowledge to improve health care. The medical ethics committee (NL 54549.091.15) approved the study protocol in December 2015. The study will start in January 2016, and is expected to finish until the end of 2018. This trail had also been registered at ClinicalTrails.gov (nr: NCT02623101).

## Discussion

Considering the increasing skin cancer problem, including BCC, and the disadvantages of the current diagnostic gold standard, histopathological diagnosis of a punch biopsy, indicates the need for cost- and time-efficient diagnostic tools with high accuracy for diagnosing skin cancers. These tools should be able to distinguish between skin cancer types and should be able to determine the correct BCC subtype, as different subtypes of BCCs are treated differently. Biopsies often result in sampling errors, as only a small part of the tumor is investigated resulting in potentially inappropriate chosen therapies. As a sample error may lead to treatment failures or recurrences, other subsequent treatments are needed. This will eventually lead to increasing costs. In addition, the conventional method is unfriendly for patients, as it is invasive, painful, and might result in scarring. Furthermore, the diagnosis cannot be made instantly.

To contribute to implementation of RCM as noninvasive skin cancer diagnostic tool, this study will investigate whether RCM is at least as good in identifying BCC subtypes as conventional histopathological investigation of skin biopsies. Hypothesizing that RCM is a cost-effective alternative to the present care, it saves on direct medical consumption like labor of the pathologist and other medical personnel as well as materials related to treatment failure with at least equal effectiveness.
